# Mobilization of CD133^+^ Progenitor Cells in Patients with Acute Cerebral Infarction

**DOI:** 10.1371/journal.pone.0070796

**Published:** 2014-03-05

**Authors:** Dominik Sepp, Daniela Franz, Natalie Triftshaeuser, Ilka Ott, Lorena Esposito-Bauer, Regina Feurer, Christian L. Seifert, Markus Thaler, Bernhard Hemmer, Holger Poppert

**Affiliations:** 1 Department of Neurology, Klinikum Rechts der Isar, Technische Universitaet Muenchen, Muenchen, Germany; 2 Department of Radiology, Klinikum Rechts der Isar, Technische Universitaet Muenchen, Muenchen, Germany; 3 Department of Cardiovascular Diseases, Deutsches Herzzentrum der Technischen Universitaet Muenchen, Muenchen, Germany; 4 Institute for Clinical Chemistry and Pathobiochemistry, Klinikum Rechts der Isar, Technische Universitaet Muenchen, Muenchen, Germany; University of California, Riverside, United States of America

## Abstract

Progenitor cells (PCs) contribute to the endogenous repair mechanism after ischemic events. Interleukin-8 (IL-8) as part of the acute inflammatory reaction may enhance PC mobilization. Also, statins are supposed to alter number and function of circulating PCs. We aimed to investigate PC mobilization after acute ischemic stroke as well as its association with inflammatory markers and statin therapy. Sixty-five patients with ischemic stroke were enrolled in the study. The number of CD133^+^ PCs was analyzed by flow cytometry. Blood samples were drawn within 24 hours after symptom onset and after 5 days. The number of CD133^+^ PCs increased significantly within 5 days (p<0.001). We found no correlation between CD133^+^ PCs and the serum levels of IL-8, IL-6, or C-reactive protein (CRP). Multivariate analysis revealed that preexisting statin therapy correlated independently with the increase of CD133^+^ PCs (p = 0.001). This study showed a mobilization of CD133^+^ PCs in patients with acute cerebral infarction within 5 days after symptom onset. The early systemic inflammatory response did not seem to be a decisive factor in the mobilization of PCs. Preexisting statin therapy was associated with the increase in CD133^+^ PCs, suggesting a potentially beneficial effect of statin therapy in patients with stroke.

## Introduction

Cerebral infarction remains a leading cause of morbidity and mortality despite recent therapeutic advances. A variety of neuroprotective approaches has been investigated in experimental studies with promising results. However, translation to clinical application has remained difficult and so far none of these approaches have been shown to be effective in clinical trials. It is thus necessary to understand the complex processes of inflammation and repair mechanisms after cerebral infarction in humans.

Circulating progenitor cells (PCs) are believed to play a key role in the concert of endogenous repair mechanisms. They support reendothelialization and neovascularization after experimental ischemic events such as myocardial infarction or limb ischemia. In 2004, in a mouse model of stroke, Taguchi et al showed an accelerated neovascularization in the ischemic zone after administration of CD34^+^ PCs that provided a favorable environment for neuronal regeneration [1]. Moreover in clinical studies patients with a higher number of PCs after ischemic stroke had a better clinical outcome, suggesting a beneficial effect [2].

The characterization and identification of the different subpopulations of PCs are still matters of controversy. The surface antigen CD34 identifies hematopoietic stem cells and progenitor cells but it is also expressed on mature endothelial cells [3], [4]. CD133 is also a progenitor cell marker but it is not expressed on mature endothelial cells and therefore can better distinguish PCs from endothelial cells. It is proven that CD133^+^ PCs can differentiate in vitro to endothelial cells [5]. Accordingly, it is supposed that CD133^+^ PCs are precursors of the more mature endothelial progenitor cells (EPCs) that already express an endothelial marker such as VEGFR (vascular endothelial growth factor receptor) [6].

The number of PCs is influenced by several factors. It is reduced in patients with cardiovascular risk factors, presumably due to decreased mobilization from the bone marrow and increased recruitment to sites of vascular injury [7], [8]. An increased mobilization of PCs has been shown during the acute phase of myocardial infarction [9]. After acute cerebral infarction divergent results have been described in previous studies. In 2009 Paczkowska et al found a higher number of CD34^+^CD133^+^ PCs in patients within 24 hours after cerebral ischemia compared with healthy subjects. No further increase after 3 and 7 days was shown [10]. In contrast Zhou et al reported that acute stroke patients presented with lower numbers of CD133^+^KDR^+^ PCs compared with healthy subjects and the number of detected PCs increased until day 7 [11]. Thus the course of PC mobilization after acute cerebral infarction remains unclear.

It is important to understand the mechanism of PC mobilization in order to be able to influence the level of PCs. Some studies suggest that inflammation may promote PC mobilization after acute ischemic events: CRP(C-reactive protein) correlates with the number of endothelial PCs in patients with unstable angina pectoris and interleukin-6 (IL-6) stimulates endothelial PCs at least in vitro [12], [13]. In a previous study in patients with acute myocardial infarction we observed interleukin-8 (IL-8) levels to be associated with circulating PCs [14]. IL-8 is a cytokine that is produced among others by macrophages, fibroblasts and endothelial cells. It influences the inflammatory process and exerts pro-angiogenic effects [15]. In experimental studies IL-8 induced stem cell mobilization by activating MMP-9 (matrixmetallopeptidase 9) and LFA-1 (lymphocyte function-associated antigen-1) [16]. Although it is known that IL-8 is increased in stroke patients, a possible correlation between IL-8 and PCs in acute cerebral infarction has not yet been analyzed [17].

The lesion volume of the cerebral infarction also seems to be correlated with the number of circulating progenitor cells. Bogoslovsky et al found higher levels of endothelial PCs in patients with a smaller volume of the acute lesion [18]. Lesion volume can be quantified by MRI or with NSE (neuron-specific enolase) as a marker of the volume of infarcted tissue, but an association between NSE and progenitor cell mobilization is not yet established.

There is little knowledge about common therapeutic agents that might influence the mobilization of PCs. HMG-CoA reductase inhibitors (statins) also seem to impact the mobilization of PCs, although the results of the published studies are conflicting. They are commonly used in stroke patients to lower cholesterol levels and as secondary prophylaxis. Whereas most studies reported an increased level of PCs after treatment with a statin, Hristov found decreased levels of PCs in patients with coronary artery disease after long-term (>8 weeks) therapy with atorvastatin or simvastatin [19], [20], [21], [22]. We found an association between preexisting statin therapy and the level of CD133^+^ PCs in patients with acute myocardial infarction [14]. However it is still unknown whether preexisting statin therapy has an impact on PC mobilization after cerebral infarction. Since long-term administration of statins is safe, with only few side effects, it could represent a promising therapeutic option to influence progenitor cell mobilization.

The aim of this study was to analyze the extent and time course of mobilization of CD133^+^ PCs after acute cerebral infarction. We further aimed to provide insight into the association with selected inflammatory markers and the potential influence of preexisting statin therapy.

## Materials and Methods

### Study population

We enrolled into the study 65 patients admitted to our stroke unit. Inclusion criteria were a clinically and radiologically proven ischemic stroke and onset of symptoms within 24 hours before admission. Exclusion criteria were chronic inflammatory, malignant, or chronic infectious diseases as well as any ischemic event including cardiac and peripheral limb ischemia within 6 months before stroke.

All subjects gave written informed consent before inclusion in the study. The study protocol was approved by the institutional local ethics committee (Ethikkommission der Fakultät für Medizin der Technischen Universität München).

Peripheral venous blood was sampled from patients within 24 hours after symptom onset (day 1) and on day 5.

We used the data of 31 patients with asymptomatic carotid artery stenosis, acquired for a previously published study, as a control group [23].

### Flow cytometry

Blood samples were processed immediately (<2 hours after blood withdrawal) for the analysis of circulating PCs. Mononuclear cells enriched from citrate phosphate dextrose acid-anticoagulated blood samples by Ficoll (Ficoll-Paque PLUS, Amersham Biosciences, Uppsala, Sweden) density gradient centrifugation were stained as previously described [14], [23]. Anti-CD133, anti-CD34, 7-AAD, and anti-CD45 were used to determine vital CD133^+^ PCs.

Fluorescence isotype-matched antibodies served as controls.

Flow cytometric analysis was performed using a FACS Calibur (Becton Dickinson, Mountain View, CA, USA). At least 100 000 events were acquired and analyzed using CellQuest software.

Antibodies used were:

PE-conjugated anti-CD133 (clone AC133) (Miltenyi Biotec, Auburn, AL, USA)
FITC-conjugated anti-CD34 (clone 8G12), 7-AAD, and APC-conjugated anti-CD45 (clone 2D1) (Becton Dickinson, Heidelberg, Germany)

### Immunoassays

Cytometric bead assays (CBA Human Inflammation Kit, BD Biosciences, San Diego, CA, USA) were used to evaluate concentrations of IL-6 and IL-8 in the blood samples on days 1 and 5.

Detection limits were 2.5 pg/mL for IL-6 and 3.6 pg/mL for IL-8. Intra-assay variability for the lower assay range was 10%.

### Statistical analysis

Differences between day 1 and day 5 were analyzed by Mann–Whitney–Wilcoxon rank-sum test (SPSS 20.0 for Windows). Linear regression analyses were performed for analyses of association (SPSS 20.0 for Windows). Results are expressed as mean (median) ± sem.

A p-value<0.05 was deemed significant.

## Results

### Clinical data

We recruited 65 patients with clinically and radiologically proven cerebral infarction. Baseline criteria and cardiovascular risk factors are shown in Table 1.

**Table 1:** Baseline characteristics.

**Characteristics**	
Age, mean (range)	68,4 (21–92)
Male, N (%)	43(66)
Hypertension, N (%)	45 (69)
Atrial fibrillation N (%)	16 (25)
Current or former smoker, N (%)	22 (34)
Hyperlipidemia, N (%)	23 (35)
Diabetes mellitus, N (%)	17 (26)
Thrombolysis, N (%)	15 (23)
**Medication**	
Aspirin, N (%)	32 (49)
Clopidogrel, N (%)	6 (9)
VKA therapy, N (%)	5 (8)
Statin, N (%)	15 (23)
ACE inhibitor, N (%)	23 (35)
ARB inhibitor, N (%)	3 (5)
Beta-blocker, N (%)	16 (25)

Abbreviations: VKA = vitamin K antagonist; ACE = angiotensin-converting enzyme; ARB = angiotensin receptor blocker.

### Time course of CD133^+^ PCs and inflammatory response

**Figure 1:**
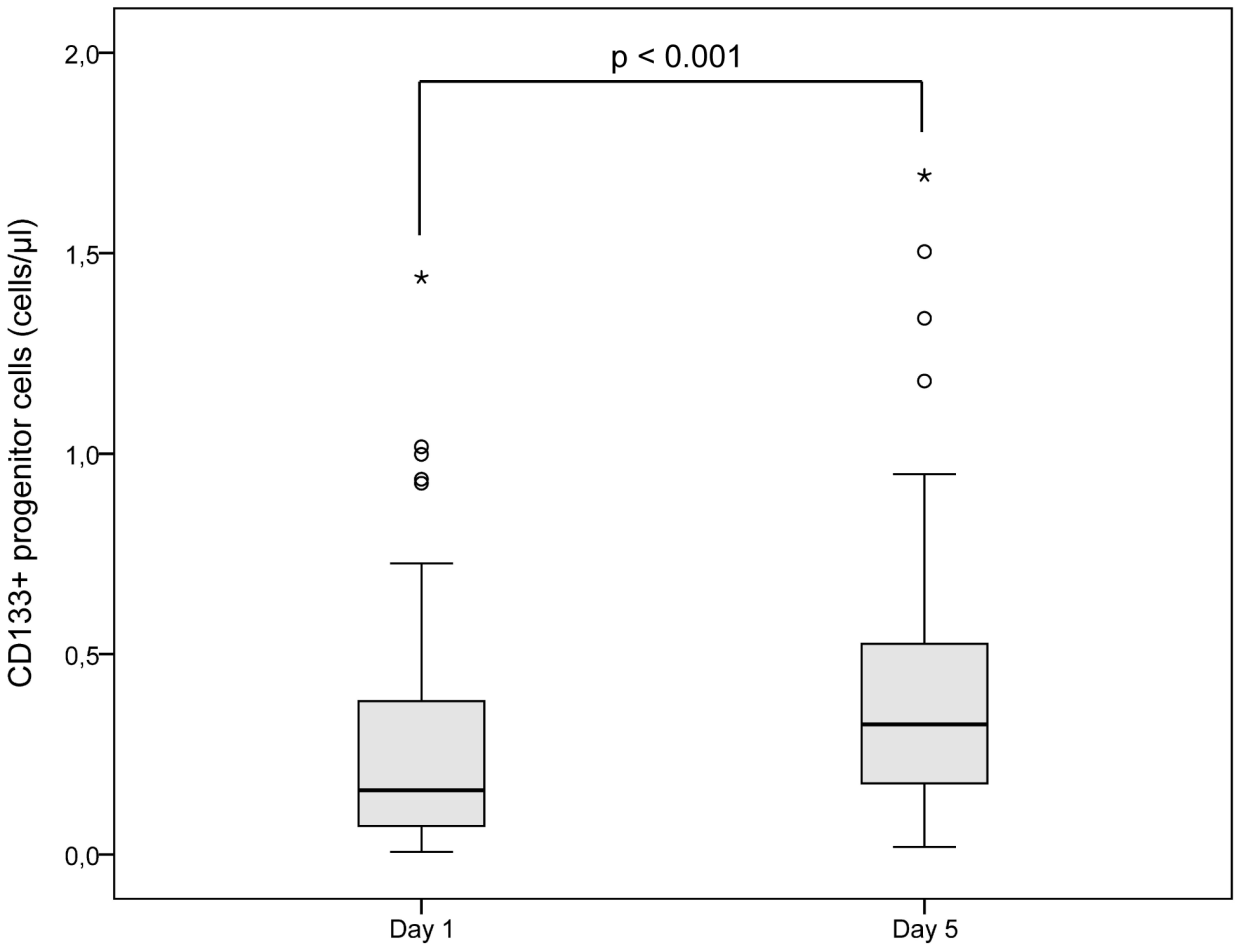
CD133^+^ progenitor cells in patients with acute cerebral infarction. Boxplot demonstrating the number of CD133^+^ progenitor cells (cells/µl) in patients with acute cerebral infarction on day 1 (within 24 hours after symptom onset) and on day 5.

The absolute number of CD133^+^ PCs increased from day 1 (within 24 hours after symptom onset) until day 5 (p<0.001) (Table 2, Figure 1). The time between symptom onset and the first blood analysis (mean = 15.15 h) did not correlate with the number of CD133^+^ PCs (p = 0.49). The control group of subjects with asymptomatic carotid stenosis had lower numbers of CD133^+^ PCs (0.13 cells/µl (0.07)±0.03) compared with stroke patients on day 1 (0.32 cells/µl (0.16)±0.07; p = 0.004).

**Table 2:** CD133^+^ PCs, inflammation parameters and NSE at day 1 and day 5.

	Day 1	Day 5	p-values
CD133^+^ PCs (cells/µl)	0.32 (0.16)±0.07	0.41 (0.32)±0.04	<0.001
Interleukin-8 (mg/dl)	21.96 (14.49)±3.42	18.06 (13)±3.42	0.45
Interleukin-6 (mg/dl)	34.5 (7.1)±12.92	12.8 (6.5)±2.47	0.12
CRP (mg/dl)	1.52 (0.4)±0.37		
Leukocytes (10^3^/µl)	8.56 (8.15)±0.42	7.43 (7.3)±0.27	0.001
NSE (µg/l)		14.53 (11.62)±1.01	

Abbreviations: PC = progenitor cell; NSE = neuron-specific enolase; CRP = C-reactive protein.

Results are expressed as mean (median) ± sem.

Concentrations of IL-8 and IL-6 did not change significantly between day 1 and day 5 (p = 0.45; p = 0.12). The number of leukocytes decreased until day 5 (p = 0.001) (Table 2).

### CD133^+^ PCs and cytokines/inflammatory parameters

Using univariate regression analysis, no significant correlation was found between the relative increase ([day5-day1]/day1) of CD133^+^ PCs and CRP(C-reactive protein), IL-8, or IL-6 (day 1). Similarly, the number of CD133^+^ PCs at admission showed no correlation with inflammatory parameters (Table 3).

**Table 3:** Univariate correlation between CD133^+^ PCs/relative increase of CD133^+^ PCs and IL-8, IL-6, NSE, number of cardiovascular risk factors, CRP and statin therapy.

	CD133^+^ PCs (day1)	Relative increase of CD133^+^ PCs ([day5-day1]/day1)
Interleukin 8 (day 1)	R = 0.04; p = 0.75	R = 0.12; p = 0.36
Interleukin 6 (day 1)	R = 0.09; p = 0.49	R = 0.16; p = 0.21
NSE (day 5)	R = 0.14; p = 0.28	R = 0.11; p = 0.39
Number of cardiovascular risk factors	R = 0.05; p = 0.67	R = 0.06; p = 0.65
CRP (day 1)	R = 0.05; p = 0.68	R = 0.19; p = 0.14
Preexisting statin therapy	R = 0.05; p = 0.68	R = 0.40; p = 0.001

Abbreviations: PC = progenitor cell; NSE = neuron-specific enolase; CRP = C-reactive protein.

### Increase of CD133^+^ PCs and preexisting statin therapy

Preexisting statin therapy was associated with a higher relative increase of CD133^+^ PCs from day 1 to day 5 (p = 0.001, R = 0.4, Table 3). The absolute number of CD133^+^ PCs on admission was not influenced by preexisting statin therapy (p = 0.68, R = 0.05). As it could be presumed that more patients with known cardiovascular risk factors were taking statins, we investigated a possible correlation between the number of cardiovascular risk factors and CD133^+^ PCs but found no significant correlation (p = 0.65). NSE (neuron-specific enolase) on day 5 as a marker of the volume of infarcted tissue did not correlate with the increase of CD133^+^ PCs (Table 3) [24].

In order to analyze whether preexisting statin medication was an independent predictor for the relative increase of CD133^+^ PCs, a multivariable analysis was performed including the following parameters presumed to influence PC mobilization: IL-8 levels on day 1 as a parameter of the inflammatory reaction; NSE levels on day 5 as a marker of the volume of infarcted tissue; and the number of cardiovascular risk factors. It was shown that preexisting statin medication is an independent predictor correlating significantly with the relative increase of CD133^+^ PCs (p = 0.001) (Table 4).

**Table 4:** Multivariate analysis between statin therapy, IL-8, number of cardiovascular risk factors, NSE, and the relative increase of CD133^+^ PCs.

	β	t	p-Values
Statin therapy	0.48	3.59	0.001
NSE	0.16	1.34	0.19
Number of cardiovascular risk factors	−0.16	−1.23	0.23
Interleukin 8	0.11	0.93	0.36

Abbreviations: PC = progenitor cell; NSE = neuron-specific enolase.

## Discussion

The study showed the following major findings. (1) The number of circulating CD133^+^ PCs increases in patients with acute cerebral infarction within 5 days after symptom onset. (2) Inflammatory parameters such as CRP, IL-6, and IL-8 on day 1 are not associated with circulating CD133^+^ PCs. (3) The relative increase, but not the initial level, of CD133^+^ PCs is higher in patients with preexisting medication with HMG-CoA reductase inhibitors (statins).

The mobilization of PCs after acute ischemic events such as myocardial infarction has already been shown in earlier studies [9], [14]. Our results concerning the increase of PCs after the ischemic event are comparable to those from these studies. It is therefore supposed that ischemic tissue influences the mobilization of PCs independently of the organ affected. So far only few studies have analyzed PCs after acute cerebral infarction. In 2004 Taguchi et al demonstrated an increase of PCs 7 days after cerebral infarction in 5 patients [25]. Further studies showed divergent results. Paczkowska et al did not find an increase of PCs (using CD133 and CD34) 3 and 7 days after cerebral infarction compared with the initial value (<24 h) [10]. In contrast, a study by Zhou et al showed an increase of PCs (using CD133 and KDR) in patients after cerebral infarction until day 7 [11]. A possible explanation of these divergent results might be that different populations of PCs were analyzed using different antibodies. Another explanation could be the different time-points of the first blood samples within the first 24 hours. Paczkowska supposed that the mobilization of PCs occurs within the first 24 hours. Our results showed a significantly higher number of CD133^+^ progenitor cells in our stroke patients on day 1 compared with the asymptomatic control group. This might indicate that, as Paczkowska suggested, the mobilization of CD133^+^ progenitor cells is initiated within the first 24 hours after cerebral infarction. However, it is known that distinct triggers such as G-CSF (granulocyte colony-stimulating factor) do not induce the mobilization of PCs until day 3, with a peak on day 5 [26]. This suggests that additional factors are influencing the mobilization after acute ischemia.

The mechanisms of mobilization of PCs are not fully understood. It is supposed that the mobilization of PCs is initiated by a vascular injury itself and by the subsequent systemic inflammatory reaction. In this study we did not find a correlation between the increase of PCs and either IL-8 or CRP/IL-6 as inflammation parameters at admission. IL-8 was correlated with the number of PCs in acute myocardial infarction in our previous study, suggesting an influence of IL-8 on the mobilization of PCs [14]. Although IL-8 is known to be increased after cerebral infarction [17], we did not find a correlation between the level of IL-8 and the increase of PCs in the present study. This could indicate that the mobilization mechanisms are different depending on the affected ischemic organ.

Therapeutic agents that influence the mobilization mechanism of PCs could represent a new therapeutic approach. HMG-CoA reductase inhibitors (statins) are supposed to play a role in the mobilization mechanism of PCs. Until now there have been few studies on the effect of statins on the number of PCs, and these have had divergent results. Vasa et al demonstrated in 2001 that atorvastatin increases the level of CD34^+^/VERGFR2^+^ PCs within one week. Subsequent studies showed similar results [19], [20], [22]. Our previously published study also observed a positive correlation between preexisting statin therapy and the level of PCs after acute myocardial infarction [14]. In contrast, Hristov et al reported a decreased number of PCs in patients with coronary artery disease after long-term therapy (>8 weeks) with statins, suggesting that the mobilizing effect of statins on PCs in the first weeks could be a transient short-term effect [21]. We did not find a significant association between preexisting statin therapy and the number of CD133^+^ PCs in our patients within the first 24 hours after acute cerebral infarction. However, we did show that patients with preexisting medication with statins had an increased mobilization of PCs until day 5 after cerebral infarction independently of other possible influencing factors such as number of risk factors and inflammatory parameters. Hence we have demonstrated a further effect of statins, which could be beneficial for patients in acute cerebral infarction. As all patients were on statins within the first week after admission, it cannot be determined by this study whether statins that are given for the first time after cerebral infarction influence the mobilization of PCs. The underlying mechanisms by which statins affect the mobilization of PCs remain unclear. Dimmeler et al proposed that statins share a common PI3K/Akt signaling pathway with growth factors such as VEGF that play a role in the PC differentiation and mobilization process [20].

In conclusion, progenitor cells are presumed to be a part of the endogenous repair mechanism and are mobilized within 5 days after acute cerebral infarction. Preexisting medication with statins is associated with augmented mobilization after infarction. This indicates that pretreatment with statins might favorably affect stroke outcome.
